# A Sporadic Giant Mesenteric Desmoid-Type Fibromatosis in a Teenage Female: A Report of a Rare Case

**DOI:** 10.7759/cureus.46087

**Published:** 2023-09-27

**Authors:** Nimah A Rabai, Arqam Alrababah, Shaima A Batayha, Saleh A Ba-shammakh, Mohammad O Sayaheen

**Affiliations:** 1 Department of General Surgery, Princess Basma Teaching Hospital, Irbid, JOR; 2 Department of Pathology, Jordan University of Science and Technology, Irbid, JOR

**Keywords:** case report, beta-catenin, mesenteric, fibromatosis, desmoid

## Abstract

Mesenteric desmoid-type fibromatosis (DTF) is a rare benign yet aggressive neoplasm that has an unpredictable biological behavior ranging from spontaneous regression to extensive local infiltration and has a high tendency for recurrence. The presenting symptoms are usually nonspecific and mostly related to the large size of the tumor compressing adjacent organs. Imaging studies can be suggestive of the diagnosis, but confirmation is based on histopathological and immunohistochemical examination. The lack of knowledge on the etiology and pathogenetic behavior of this tumor leads to therapeutic and prognostic challenges. Future genetic studies may help in advancing our understanding of this neoplasm and in formulating the proper management and follow-up plan. Here we present a case of a 14-year-old female who presented to the emergency room complaining of diffuse abdominal pain and distention. A computed tomography (CT) scan showed a large mass occupying most of the abdominal cavity and compressing adjacent organs. Exploratory laparotomy with resection and anastomosis was performed, and the histopathological and immunohistochemical examination of the resected mass was consistent with mesenteric DTF.

## Introduction

Mesenteric desmoid-type fibromatosis (DTF) is an infrequent benign yet invasive tumor, known for its potential to infiltrate locally and recur, even following surgical removal [1]. It represents less than 1% of abdominal tumors and has a peak incidence between 20 and 40 years of age, with female preponderance [2,3]. The etiology of this neoplasm is not well known yet. It has been associated with hereditary diseases like familial adenomatous polyposis (FAP) and Gardner syndrome, with some physical and hormonal changes like trauma, surgery, and excess estrogen state [2]. Patients with this tumor usually present with nonspecific symptoms related to the large size of the tumor that grows insidiously causing compression and displacement of adjacent structures. Imaging studies can help in making the diagnosis, but histopathological and immunohistochemical examination is the standard method to confirm the diagnosis [2]. Herein, we report a case of a giant mesenteric DTF in a 14-year-old female, who was not found to have any predisposing factor. Due to the large size and the symptoms of the patient, a decision was made to resect the mass with the involved small bowel and to do primary anastomosis. The diagnosis was confirmed with histopathological and immunohistochemical examination and was consistent with mesenteric DTF.

## Case presentation

A 14-year-old female patient, previously healthy, presented to the emergency room (ER) complaining of diffuse abdominal pain for two days. The pain was gradual in onset and colicky in nature, progressive in severity over time, associated with vomiting five times a day, containing ingested food and gastric juice, with no blood, no coffee ground vomitus, and no bile. It was also associated with a fever of 38°C, in a background of chronic constipation and abdominal distention for a few months. The patient stated that the abdominal distention started a couple of months earlier when she simply assumed she was getting obese and decided to follow a very strict diet to lose weight. Consequently, she developed symptoms of dizziness and palpitation, in addition to irregularity in her menstrual cycle, which could be related to anemia. Her past medical history was free, with no previous diagnosis of colon polyps or any other colon disease. She had no previous surgeries, including abdominal, and no history of trauma. She was not on any medications and had no allergies. Her family history was free, especially of colon polyps, FAP, or Gardner’s syndrome.

On physical examination, the patient was conscious, alert, and oriented, and her Glasgow Coma Score (GCS) was 15/15. She looked pale, in pain, with tachycardia of 133 beats per minute (bpm) but normal blood pressure was 134/74 mm Hg. Her temperature was 38.4°C orally, and her oxygen saturation was 98% in room air.

The abdomen was diffusely distended and tender all over. It was dull to percussion with no fluid thrill. Bowel sounds were audible and her digital rectal examination (DRE) was normal with soft stool present in the rectum and no palpable abnormalities.

Her lab workup was consistent with anemia with mild elevation of liver enzymes (Table 1).

**Table 1:** The patient's lab workup on admission. CBC, complete blood count; WBC, white blood cells; HgB, hemoglobin; HCT, hematocrit; MCV, mean corpuscular volume; PLT, platelets; INR, international normalized ratio; CPK, creatine phosphokinase; ALP, alkaline phosphatase; AST, aspartate aminotransferase; ALT, alanine aminotransferase; Na+, sodium; K+: potassium

Parameter	Value	Reference Range
CBC		
WBC	8.82 x 10^3^ /uL	4.0-11.0 x 10^3^/uL
HgB	8.0 g/dL	13.8-17.2 g/dL for males, 12.1-15.1 g/dL for females
HCT	25.7%	38.3-48.6% for males, 35.5-44.9% for females
MCV	60.9 fL	80-96 fL
PLT	304 x 10^3^/uL	150-400 x 10^3^/uL
Coagulation		
INR	1.07	~0.8-1.2
Blood Chemistry		
Glucose	5.54 mmol/L	3.9-6.1 mmol/L
Urea	2.1 mmol/L	2.5-6.7 mmol/L
Creatinine	46 umol/L	59-104 umol/L for males, 45-90 umol/L for females
CPK	66 U/L	39-308 U/L
Amylase	37 U/L	30-110 U/L
Total Bilirubin	10.8 umol/L	5.1-20.5 umol/L
Direct Bilirubin	7.0 umol/L	0-5.1 umol/L
ALP	101 U/L	45-117 U/L
AST	78.1 U/L	0-37 U/L
ALT	100.9 U/L	0-42 U/L
Sodium (Na+)	131 mmol/L	136-145 mmol/L
Potassium (K+)	4.02 mmol/L	3.5-5.1 mmol/L

**Figure 1:**
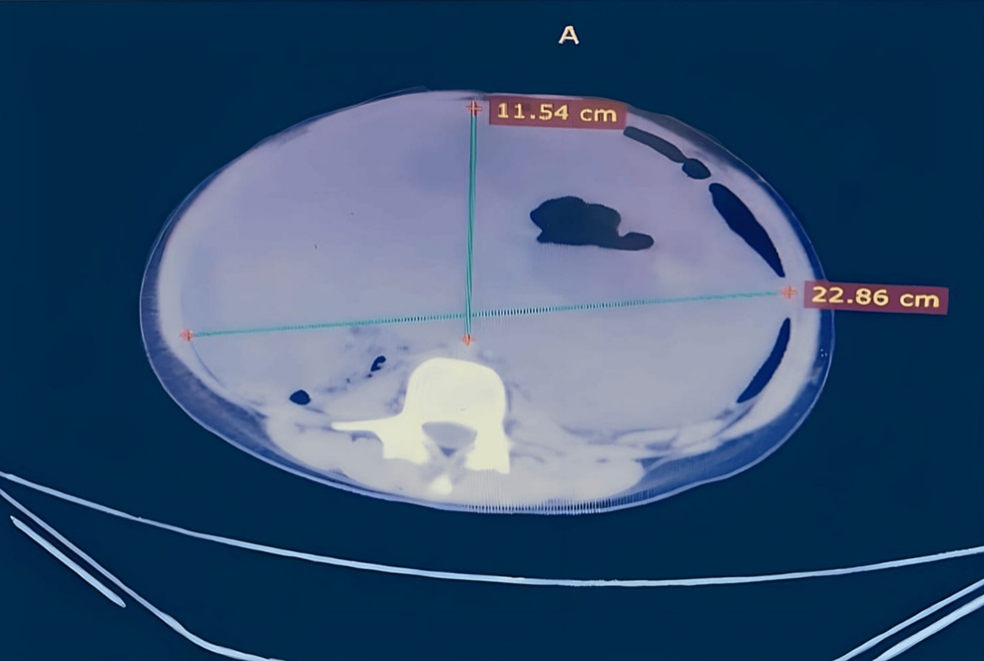
CT scan with IV contrast shows a large lobulated soft tissue mass with internal cystic component occupying the abdominal cavity and causing compression of adjacent organs and blood vessels. CT, computed tomography

The patient underwent computed tomography (CT) scan with IV contrast; the CT report stated that there is a large lobulated soft tissue mass with an internal cystic component occupying the abdominal and pelvic cavities, measuring approximately 30x20x10 cm, causing compression of adjacent organs and blood vessels with lateral displacement of these structures (Figure 1).

The mass shows minimal enhancement on delayed post-contrast imaging. These findings were suggestive of mesenteric desmoid tumor, lymphoma, ovarian cystadenoma, or ovarian cancer.

Resuscitation with IV fluids (Lactated Ringer) was initiated in addition to prophylactic IV antibiotics (cefuroxime and metronidazole) and analgesic (IV paracetamol). Blood group and cross-match were tested and preparation of blood components including packed red blood cells (pRBCs) and plasma was requested. Two units of cross-matched pRBCs were given preoperatively.

Due to the large size of the mass and the symptoms caused by mass pressure on adjacent organs, a decision was made with the patient and her family to perform surgical exploration with resection.

**Figure 2:**
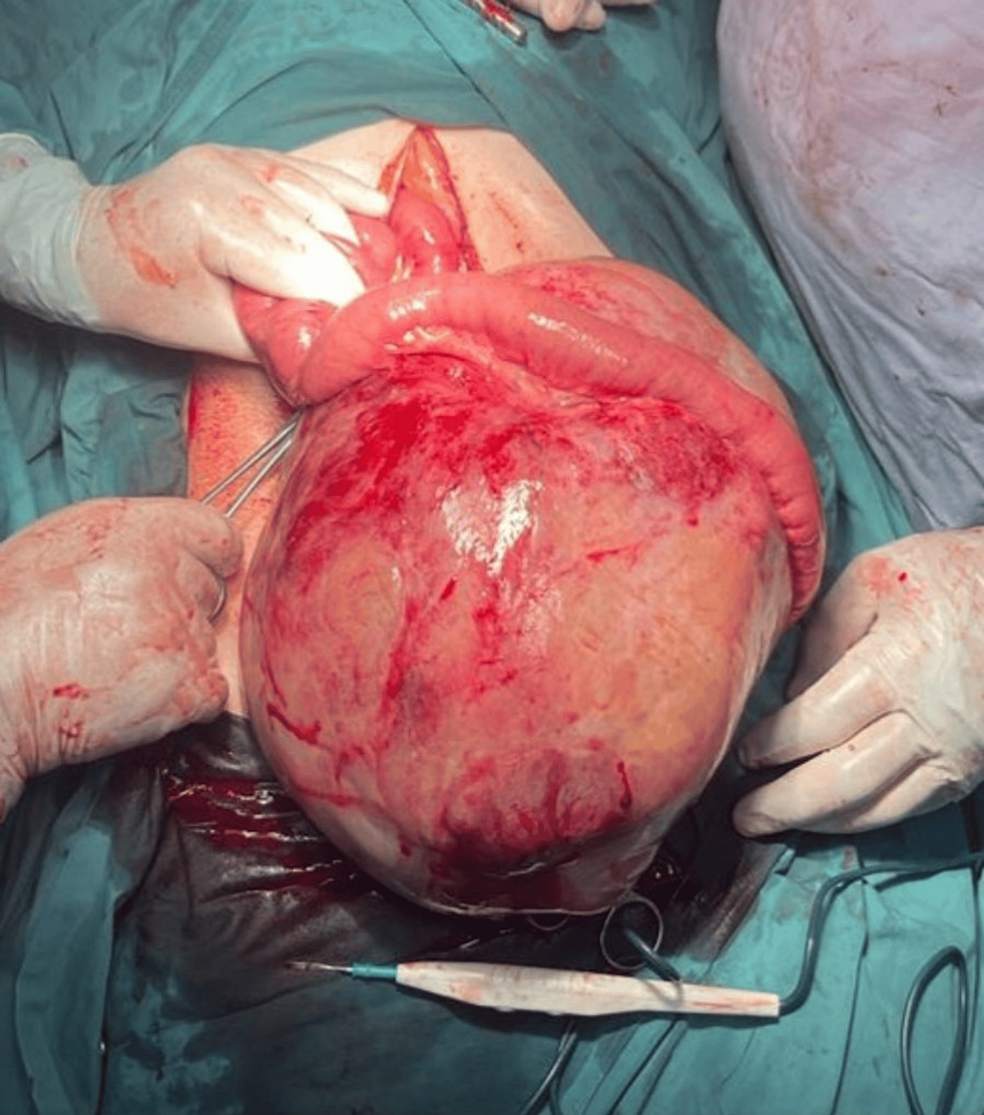
Intraoperative image of the mass.

**Figure 3:**
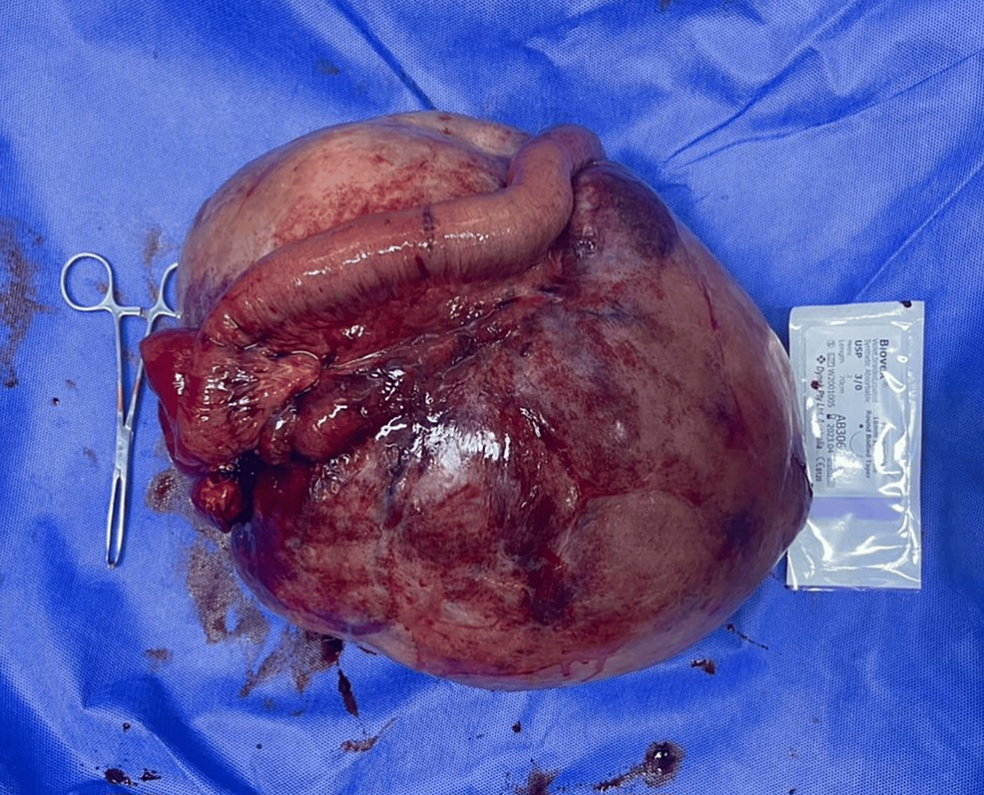
The resected mass with the attached segment of the small bowel.

The patient underwent exploratory laparotomy. After the incision was made, and layers opened down to the peritoneum; a large mass was immediately appreciable, occupying most of the abdominal cavity, from the pelvic region to the epigastric area. The mass is contained within the mesentery of the small bowel, specifically the mesentery supplying the right side of the bowel, causing mass effects on both the small and large bowel and other abdominal organs. The involved mesentery was found extending from the mid ilium to the midpoint of the ascending colon, with the mass abutting the margins of the bowel along these areas. A decision was made to resect the mass with the attached bowel (Figure 2 and Figure 3).

After resecting the mass, a primary end-to-end ileocolic anastomosis was performed with the closure of the mesenteric defect. After securing hemostasis and confirming the correct count of towels and instruments, a pelvic drain was inserted and the incision was closed layer by layer.

On histopathological examination of the mass, the mass grossly measured 30x21x11 cm with 66 cm of attached bowel. The cut surface was pink with the site of cystic formation and hemorrhage. The sample also included the resected bowel with an attached appendix measuring 6.5x1x1 cm. The sample contained two large lymph nodes.

**Figure 4:**
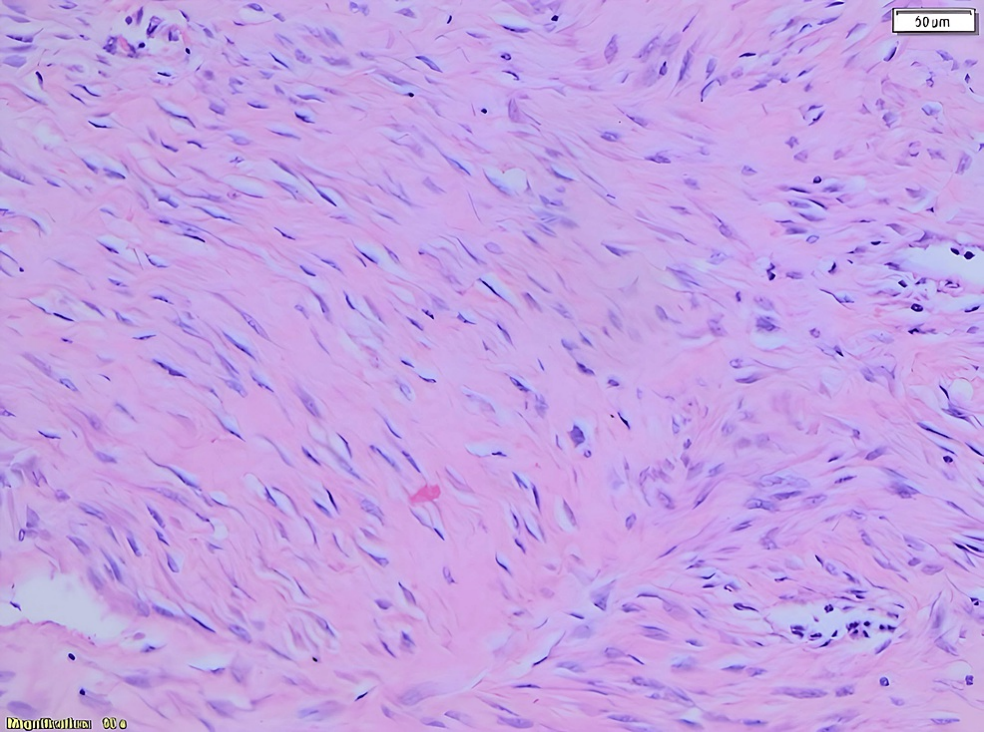
Long sweeping fascicles with elongated, slender, spindled cells of uniform appearance and pale cytoplasm set in a collagenous stroma. Magnification 10x

Microscopically, the mass was found to contain mesenteric large spindle cell neoplasm with bland-looking nuclei. The lesion had pushing growth for the most part, but focally tumor cells intersect with the muscle part of the bowel wall (Figure 4).

**Figure 5:**
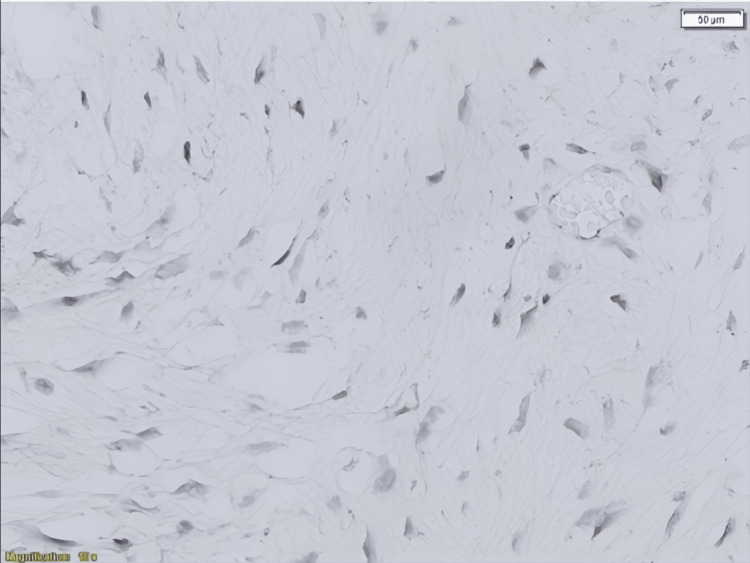
Beta-catenin immunostain shows nuclear positivity. Magnification 10x

On immunohistochemical examination, the lesion cells had nuclear immunoactivity for beta-catenin immunostain but were negative for S100, CD34, Desmin, and DOG1 (Figure 5).

The diagnosis was consistent with mesenteric deep fibromatosis (desmoid-type) with unremarkable overlying epithelium and unremarkable appendix. The total lymph nodes harvested within the sample were five and found to be reactive lymph nodes.

The patient had a prolonged recovery. She had recurrent attacks of abdominal pain despite receiving regular analgesics. Her follow-up evaluation, lab workup, and imaging did not identify any possible intra-abdominal surgical complication. After the post-operative day 5, there was a minimal discharge from the distal end of the incision, which was treated with drainage and dressing, but further delayed the recovery progress.

The patient was discharged on post-operative day 13 with stable vital signs, cessation of abdominal pain, toleration of oral intake including solid food, normal bowel motion, and clean operative wound. After discharge, she was referred to the oncology clinic to continue the proper medical management of her condition. As she underwent complete resection, her oncologist decided to follow her up and perform yearly imaging surveillance without using any medical treatment. Over a year of follow-up, no recurrence or complications have been reported so far, and her surveillance CT scan was normal.

## Discussion

DTF within the abdominal region is a rare yet assertive benign tumor, accounting for a scant 0.03% of all tumors, predominantly occurring in the bowel's mesentery [3-4]. Typically affecting adults between 20 and 40, mesenteric DTF presents a notable gender disparity: women have a two- to three-fold higher likelihood of encountering this neoplasm than men [3,5]. The patient we present herein was a female of 14 years of age.

Etiology

The etiological factors predisposing to this tumor are not well known yet [4,6]. Multiple factors play a role in the pathogenesis of DFT, including physical, hormonal, and genetic factors [7]. It can occur in either hereditary or sporadic patterns. Hereditary cases are associated with FAP and Gardner syndrome, which have a high risk of abdominal DTF. On the other hand, sporadic cases rarely occur in the abdomen, representing only 5% of the cases and are commonly associated with some risk factors, including female gender, previous abdominal trauma or surgery, or high estrogen state due to hormonal treatment or pregnancy and childbirth [3]. Most published cases of mesenteric DTF had a preceding history of surgery or abdominal trauma [4].

The patient we present herein was a young female who had no personal or family history of FAP or Gardner syndrome, no history of trauma or abdominal surgeries and was not on any hormonal treatment.

Presentation

Most of the symptoms caused by mesenteric DTF are nonspecific and commonly related to mass effect, as these tumors increase in size unnoticeably before becoming problematic causing pressure on adjacent organs including the bowel, solid organs, or vascular structures [4]. As highlighted in a study documented by Elisa Palladino and colleagues, there was an instance where a patient experienced acute pulmonary embolism due to the compression of the inferior vena cava by an expansive mesenteric desmoid tumor [6].

Patients with this tumor may complain of abdominal pain of a variable course, the subacute or chronic, abdominal mass that could be felt by the patient, fever, symptoms of bowel obstruction, bowel ischemia, gastrointestinal bleeding, or hydronephrosis among others [1,5,8].

Our patient presented with subacute abdominal pain and distention that increased in severity over a few months and was associated with constipation and fever.

Diagnosis

Imaging modalities including ultrasonography (US), CT, and magnetic resonance imaging (MRI) scans can help in making the diagnosis, determining the resectability of the tumor, and planning the proper surgical procedure if the tumor is found to be resectable.

US scan is frequently the first imaging modality to use, due to its low cost and availability. It can detect a soft tissue mass, with or without cystic components, yet it does not show specific radiological features of abdominal DTF [3].

Of the imaging modalities available, a CT scan with contrast is the scan of choice for DTF. Masses can appear solitary or multiple, they may be homogenous or heterogenous, solid or cystic or both, they can be well-defined or ill-defined with infiltration to adjacent organs, and the radiological signs related to the mass effect or infiltration to adjacent organs will be evident, including signs of bowel obstruction or ischemia, vascular obstruction, hydronephrosis or solid organ compression and displacement [5].

MRI scan defines the soft tissue characteristics and morphology in better quality compared to CT scan, providing more data regarding the degree of cellularity and presence of necrosis within the mass, but due to cost and availability issues, in addition to the relatively longer time needed to conduct the scan, it is the not the standard scan for imaging, especially in emergency cases [5].

Despite having some specific features on imaging, abdominal DTF can radiologically mimic other tumors including GIST. So, the diagnosis is confirmed with histopathological examination and immunohistochemical staining of the biopsy samples or the resected mass [5].

Histologically, DTF is composed of spindle or stellate fibroblasts within a collagen stroma. Examination of the periphery might give clues to the presence of infiltration or the lack thereof [8]. On immunohistochemical examination, DTF usually expresses alpha-smooth muscle actin and beta-catenin. The positive reaction to these stains, especially beta-catenin, helps differentiate DTF from other radiological mimickers like GIST, leiomyoma, leiomyosarcoma, neurofibromas, and others [9].

Genetic studies have a promising role in diagnosing DTF through the detection of specific mutations in the tumor and help guide the choice of therapeutic intervention. It has been reported that using next-generation mutation analysis sequencing for detecting CTNNB1 mutation in sporadic DTF can reach a sensitivity of 92% and a specificity of 100%. Consequently, this can guide treatment with COX 2 selective inhibitors [2]. So far, studies identified three major mutations in CTNNB1 associated with sporadic DTF [10]. The hereditary types of DTF, specifically FAP and Gardner’s related, have mutations in the APC tumor suppressor gene that activates the tumorigenesis of DTF through loss of regulation of beta-catenin/wnt growth factor pathway, without the involvement of a mutated CTNNB1 gene [1-2].

Differential diagnoses include GIST, carcinoid tumors, intestinal tumors including carcinoma, mesothelioma, lymphoma, large hematoma, and fibrosarcoma [1,5-6].

Management

Treatment of mesenteric DTF is controversial. A period of watchful observation with active surveillance is advised initially for some cases when the presentation is non-fatal, and the patient’s status and the tumor’s characteristics permit a period of “watchful waiting.” This is based on the observation that some tumors regress spontaneously without any treatment, while complete resection of the tumor is widely considered the standard treatment for resectable tumors, especially when symptomatic, as we performed in the case we present herein [6,11-13]. Resection should aim to remove the mass with clear margins without adversely affecting involved vital structures. This can be technically difficult to achieve in mesenteric DTF in comparison to extra-abdominal DTF, as these tumors usually involve the bowel and its blood vessels or other contiguous structures, which makes extensive resection a risky procedure associated with a higher rate of morbidity and mortality [14]. Still, failure to completely resect the mass might lead to a high recurrence rate, so resection with negative margins can reduce the rate of recurrence to 27% in comparison to 54% in cases with positive margins of resection [15]. The overall incidence of recurrence reaches 40% and it is especially higher in the first two years following resection [4,12,15]. Other risk factors that may increase the risk of recurrence include aggressive type of tumor, multicentric lesions, and FAP-related mesenteric DTF; it is advisable to provide imaging surveillance for these patients for up to 3 years using CT or MRI scan, then with US scan thereafter to detect any recurrence early on [4,5,14].

The fact that there are reported cases of recurrence after complete resection signifies the aggressiveness of this tumor and the dilemma in the management of this condition and necessitates a better understanding of the tumor and its pathological course [8].

Other treatment modalities are explored, particularly in cases where tumors are inoperable, demonstrate aggressive characteristics, or recur. The effectiveness of these treatments can vary and encompass a range of medical options such as nonsteroidal anti-inflammatory drugs (NSAIDs), tamoxifen, radiation therapy, chemotherapy involving cytotoxic agents, and sorafenib, a type of tyrosine kinase inhibitor [1,14,16]. These treatments might be utilized alone or in conjunction with surgical procedures.

The variability associated with mesenteric DTF’s presentation, location, and size and our limited understanding of the possible etiological causes and course of progression of DTF have led to therapeutic and prognostic challenges in treating this condition [6]. Future genetic studies exploring the genetic makeup of the tumor and the associated mutations might help in advancing our knowledge regarding the biological behavior of this tumor and in formulating more efficient therapeutic agents to treat this pathology [1].

## Conclusions

Mesenteric DTF is a rare benign tumor that is notorious for being aggressive and recurrent. It usually presents with symptoms related to the mass effect caused by the large size of the tumor, and it should be considered in the differential diagnosis of a large abdominal mass. Data is still lacking regarding the etiological factors leading to DTF, which limits our knowledge of the prognosis and risk of recurrence, as well as the appropriate treatment plan to implement. Therefore, management should be individualized based on the clinical, radiological, and pathological findings.

Future genetic investigations are needed to further understand the biological course of this tumor, which would eventually help clinicians better manage this condition in both the short and long terms.

## References

[1] Sioda NA, Wakim AA, Wong T, Patel S, Coan K, Row D (2020). A large sporadic intra-abdominal desmoid-Type fibromatosis in a young male: a case report. Front Surg.

[2] Basim A, Patrick S, O'Dwyer ST (2021). Desmoid fibromatosis presenting as an omental mass: a case report. Arch Clin Med Case Rep.

[3] Kovačević K, Obad-Kovačević D, Popić-Ramač J (2017). Sporadic giant intra-abdominal desmoid tumor: a radiological case report. Mol Clin Oncol.

[4] Sullivan JL, Chesley PM, Nguyen DT (2022). Mesenteric desmoid tumor: De novo occurrence or recurrence following appendectomy?. Radiol Case Rep.

[5] Patel S, Jain J (2019). CT imaging features of abdominal (mesenteric) desmoid tumor. Eurorad.

[6] Palladino E, Nsenda J, Siboni R, Lechner C (2014). A giant mesenteric desmoid tumor revealed by acute pulmonary embolism due to compression of the inferior vena cava. Am J Case Rep.

[7] Casadei M, Ricci S, Sergi W, Bollino R, Pucciatti I, Bonilauri S (2021). Desmoid-type fibromatosis of the mesentery with multiple localization presenting as acute abdomen: two rare case reports in emergency surgery. Int J Case Rep Images.

[8] Faria SC, Iyer RB, Rashid A, Ellis L, Whitman GJ (2004). Desmoid tumor of the small bowel and the mesentery. AJR Am J Roentgenol.

[9] Li Destri G, Ferraro MJ, Calabrini M, Pennisi M, Magro G (2014). Desmoid-type fibromatosis of the mesentery: report of a sporadic case with emphasis on differential diagnostic problems. Case Rep Med.

[10] Shafi S, Patton A, Rogers A, Grignol V, Oghumu S, Iwenofu OH (2022). Hans Iwenfu, sporadic mesenteric desmoid-type fibromatosis with “double-hit” T41a and S45p beta-catenin mutation profile: a case report of an extremely rare event-clinically relevant or much ado about nothing?. Precis Cancer Med.

[11] Chang T, Sa T, Yu M, Zhang B, Lyu Z (2022). Gas-containing mesenteric desmoid-type fibromatosis: a case report. Medicine (Baltimore).

[12] Zhang Z, Shi J, Yang T, Liu T, Zhang K (2021). Management of aggressive fibromatosis. Oncol Lett.

[13] Sugrue JJ, Cohen SB, Marshall RM, Riker AI (2015). Palliative resection of a giant mesenteric desmoid tumor. Ochsner J.

[14] Smith AJ, Lewis JJ, Merchant NB, Leung DH, Woodruff JM, Brennan MF (2000). Surgical management of intra-abdominal desmoid tumours. Br J Surg.

[15] Tamura K, Tani M, Kinoshita H, Yamaue H (2006). Mesenteric desmoid tumor of the interposed jejunal pouch after total gastrectomy. World J Surg Oncol.

[16] Atanassova AY, Chakarov SS (2017). Intra-abdominal mesenteric desmoid tumors (DTS) after kidney transplantation: a case report. J Gastrointest Disord Liver Func.

